# Tunable superconducting neurons for networks based on radial basis functions

**DOI:** 10.3762/bjnano.13.37

**Published:** 2022-05-18

**Authors:** Andrey E Schegolev, Nikolay V Klenov, Sergey V Bakurskiy, Igor I Soloviev, Mikhail Yu Kupriyanov, Maxim V Tereshonok, Anatoli S Sidorenko

**Affiliations:** 1 Skobeltsyn Institute of Nuclear Physics, Lomonosov Moscow State University, 119991 Moscow, Russia; 2 Moscow Technical University of Communication and Informatics (MTUCI), 111024 Moscow, Russia; 3 Faculty of Physics, Lomonosov Moscow State University, 119991 Moscow, Russia; 4 Lobachevsky State University of Nizhni Novgorod Faculty of Physics, 603950 Nizhny Novgorod, Russia; 5 Dukhov All-Russia Research Institute of Automatics, 101000 Moscow, Russia; 6 Institute of Electronic Engineering and Nanotechnologies ASM, MD2028 Kishinev, Moldova; 7 Laboratory of Functional Nanostructures, Orel State University named after I.S. Turgenev, 302026, Orel, Russia

**Keywords:** networks on radial basis functions, Josephson circuits, radial basis functions (RBFs), spintronics, superconducting electronics, superconducting neural network

## Abstract

The hardware implementation of signal microprocessors based on superconducting technologies seems relevant for a number of niche tasks where performance and energy efficiency are critically important. In this paper, we consider the basic elements for superconducting neural networks on radial basis functions. We examine the static and dynamic activation functions of the proposed neuron. Special attention is paid to tuning the activation functions to a Gaussian form with relatively large amplitude. For the practical implementation of the required tunability, we proposed and investigated heterostructures designed for the implementation of adjustable inductors that consist of superconducting, ferromagnetic, and normal layers.

## Introduction

For modern telecommunications, probabilistic identification of various sources in a broadband group signal is extremely important. Also, probabilistic analysis is used in the consideration of stochastic processes [1–4], as a popular machine learning method for spatial interpolation of non-stationary and non-Gaussian data [5], as a central part of a compensation block to enhance the tracking performance in control systems for a class of nonlinear and non-Gaussian stochastic dynamic processes [6].

An important example for this work is the cognitive radio, which is able to receive information about the features of the “radio environment” and adjust its operating parameters based on this data [7–13]. Similar problems arise nowadays when reading data in superconducting noisy intermediate-scale quantum (NISQ) computers [14–17]. Here again, we need real-time identification and classification of varying signals from multiple sources (qubits) in a narrow frequency range. When working with large data, it is necessary to create specialized neural networks at the hardware level to effectively solve such problems.

Josephson digital circuits and analog receivers have been used for a long time to create software-defined radio-systems [18–25] as well as read-out circuits for quantum computing [26–33]. They realize a unique combination of a wide dynamic range and high sensitivity when receiving signals, with high performance and energy efficiency at the stage of the processing. It seems reasonable to implement additional processing of incoming data inside the cryosystem using the capabilities of neural network computing [34–43]. The creation of an extremely low-dissipating element base for such systems is a very actual scientific and technical task, which requires theoretical and experimental studies of the features of macroscopic quantum interference in the complex Josephson circuits.

The direct use of the previously proposed superconducting adiabatic neural network (ANN) based on the perceptron [44–48] for probabilistic identification is not possible. In particular, during the formation of the output signal in the ANN, the so-called global approximation of the input signal is implemented [11–12], in which almost all neurons are included in signal processing. In addition, the perceptron is a fully connected network, which means an abundance of synaptic connections between neurons. These circumstances suppose a highly resource-intensive learning of the network for signal analysis. There is an alternative approach with a representation of the input set of data into the set of output values by using only one hidden layer of neurons. Each of these neurons is responsible for its own area of the parameter space of incoming data. This is the probabilistic or Bayesian approach, where radial basis functions (for example, Gaussian-like functions) are used as neuron activation functions.

The most common networks operating on this principle are radial basis function networks (RBFNs) (also known as Bayesian networks). When using such a network, objects are classified on the basis of assessments of their proximity to neighboring samples. For each sample, a decision can be made based on the selection of the most likely class from those to which the sample could belong. Such a solution requires an estimate of the probability density function for each class. This score is obtained by consideration of training data. The formal rule is that the class with the tightest distribution in the scope of the unknown instance will take precedence over other classes. The traditional approach for estimating the probability density for each class is to assume that the density has some definite form. The normal distribution is the most preferred since it allows one to estimate such parameters of the model as the mean and standard deviation analytically. The superconducting implementation of the key elements of the discussed neural networks is the focus of this work.

## Results and Discussion

### Model of tunable Gauss-neuron: numerical simulations

**Figure 1:**
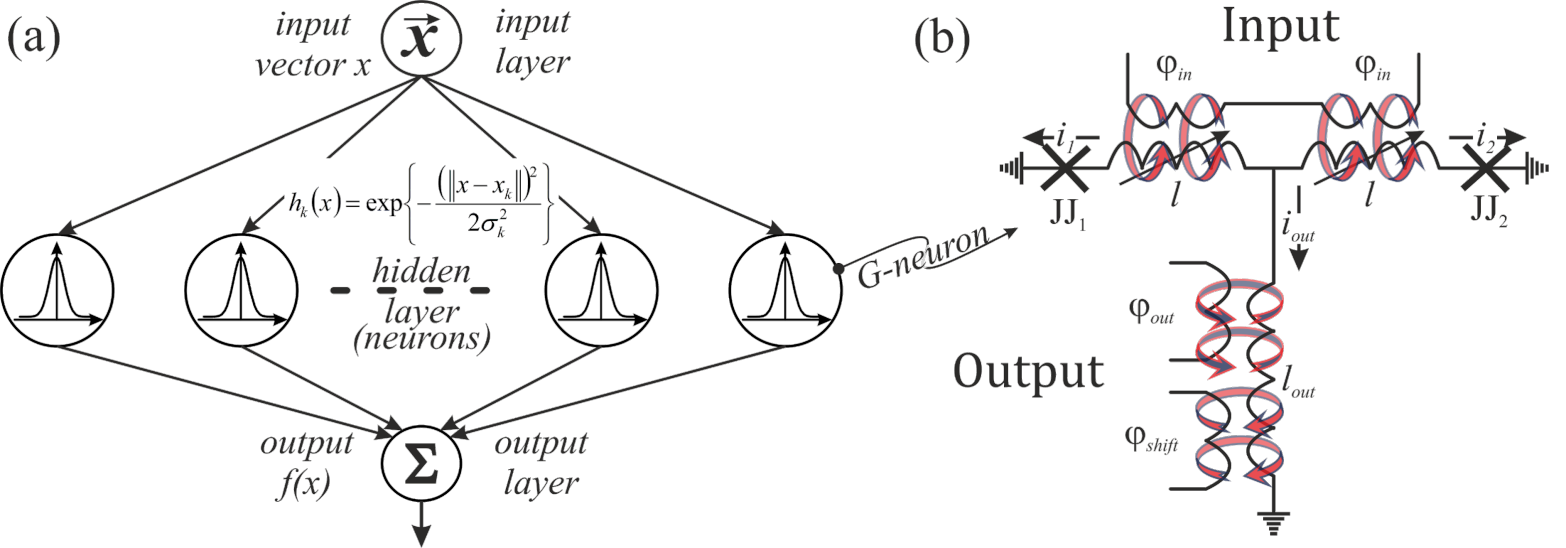
(a) Schematic illustration of a RBF network. (b) Schematic representation of a Gauss-neuron ensuring a Gauss-like transfer function.

A common architecture of the considered RBFNs [49] is presented in Figure 1a. These networks have only one hidden layer of neurons on which components of the input vector *x* are fed. Every neuron of the hidden layer calculates the values of the 1D function *h_k_*(*x*).


[1]

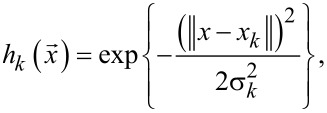



where *x_k_* is the *k*-th reference point and σ*_k_* is the scattering parameter for the one-dimensional function 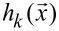.

In this paper, we propose a modified tunable neuron circuit [44] for RBFNs (see Figure 1b), with a Gaussian-like activation function. It consists of two identical Josephson junctions JJ_1_ and JJ_2_ in the shoulders with input inductances, *L*, and output inductance *L*_out_. It is also used to set an additional bias magnetic flux, Φ_b_. Flux biasing is used to provide a suitable transfer function for asynchronous circulation of currents in the connected circuits. In the following, we will call such a cell a “Gauss-neuron” or a “G-cell/neuron”.

Hereinafter, we use normalized values for typical parameters of the circuit. All fluxes (input Φ_in_ and output Φ_out_, and bias Φ_b_) are normalized to the flux quantum Φ_0_; currents are normalized to the critical current of the Josephson junctions *I*_C_; inductances are normalized to the characteristic inductance 2π*LI*_C_/Φ_0_, times are normalised to the characteristic time *t*_C_ = Φ_0_/(2π*V*_C_) (*V*_C_ is the characteristic voltage of a Josephson junction). Equations of motion were obtained in terms of half-sum and half-difference of Josephson phases φ_1_, φ_2_ (θ = (φ_1_ + φ_2_)/2 and ψ = (φ_1_ − φ_2_)/2), a detailed derivation of the equations is given in the Appendix section:


[2]

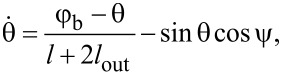




[3]

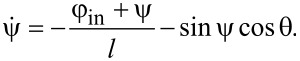



The output magnetic flux obeys the following equation:


[4] $$\varphi_{\text{out}}=\frac{2l_{\text{out}}}{l+2l_{\text{out}}}\cdot \left(\theta -\varphi_{\text{b}}\right).$$

**Figure 2:**
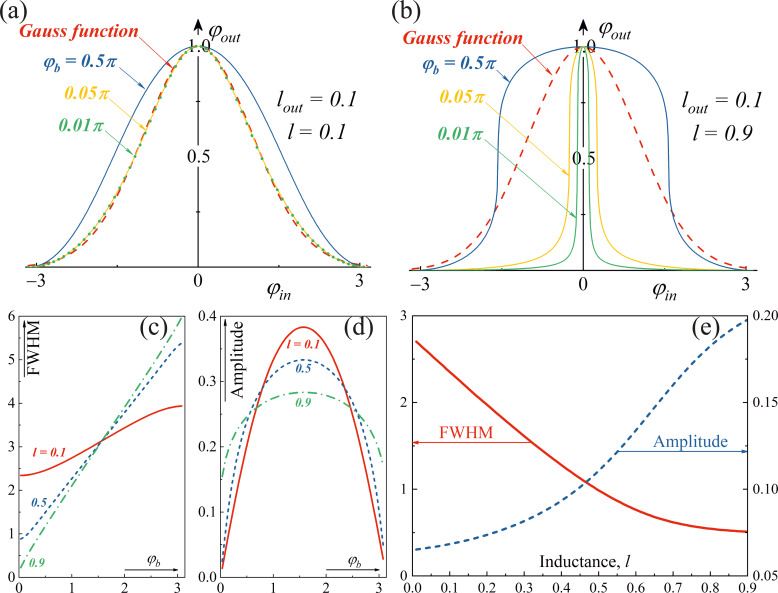
Transfer functions (normalized) and their main characteristics for the Gauss-neuron. (a, b) Families of the normalised transfer functions depending on the magnitude of the bias flux φ_b_ for various pairs of inductances *l* and *l*_out_: (a) *l* = 0.1, *l*_out_ = 0.1; (b) *l* = 0.9, *l*_out_ = 0.1. (c) Dependencies of FWHM and amplitude on the bias flux φ_b_ of transfer functions for *l* = 0.1, 0.5, and 0.9 with *l*_out_ = 0.1. (d) Dependencies of FWHM and amplitude on the inductance *l* for transfer functions of the Gauss-neuron at *l*_out_ = 0.1 and φ_b_ = 0.05π.

Figure 2a,b shows the families of transfer functions of a Gauss-neuron at different bias fluxes. They are compared with the radial basis function taken in the form *g*(*x*) = exp(−*x*^2^/(2σ^2^)) (dashed line). All transfer functions were normalized to their maximum value, since at the first stage we were interested in the shape of the curve itself. It can be seen that the shape of the response meets the requirements; in addition, it can be adjusted using a bias magnetic flux φ_b_. An important feature of the system is that it also allows for non-volatile tuning with memory using tunable inductances *l* and *l*_out_, see Figure 2c–e. Estimations for different values of φ_b_ show that the best match (with Gauss-like radial basis function) can be achieved with φ_b_ = 0.05π and inductance values of *l* = 0.1 and *l*_out_ = 0.1. Also the investigation of the full width at half maximum (FWHM) and of the amplitude of the transfer functions of the Gauss-neuron was carried out for different values of φ_b_ (Figure 2c,d) and inductance *l* (Figure 2e). It can be seen that an increase in the value of the inductance *l* decreases the FWHM of the transfer function and increases its amplitude. The bias flux is a convenient adjustment of the transfer function of the tunable Gauss-neuron; the bias flux should vary in the [0…0.5]π range to save the proper form of the transfer function. The mean of the transfer function can be controlled by an additional constant component in the input flux. By selecting the parameters of a configurable G-neuron, we can make the effective field period for the activation function (resulting from the Φ_0_-periodicity of all flux dependencies for the interferometer-based structures) large enough for practical use in real neural networks (Figure 2e).

**Figure 3:**
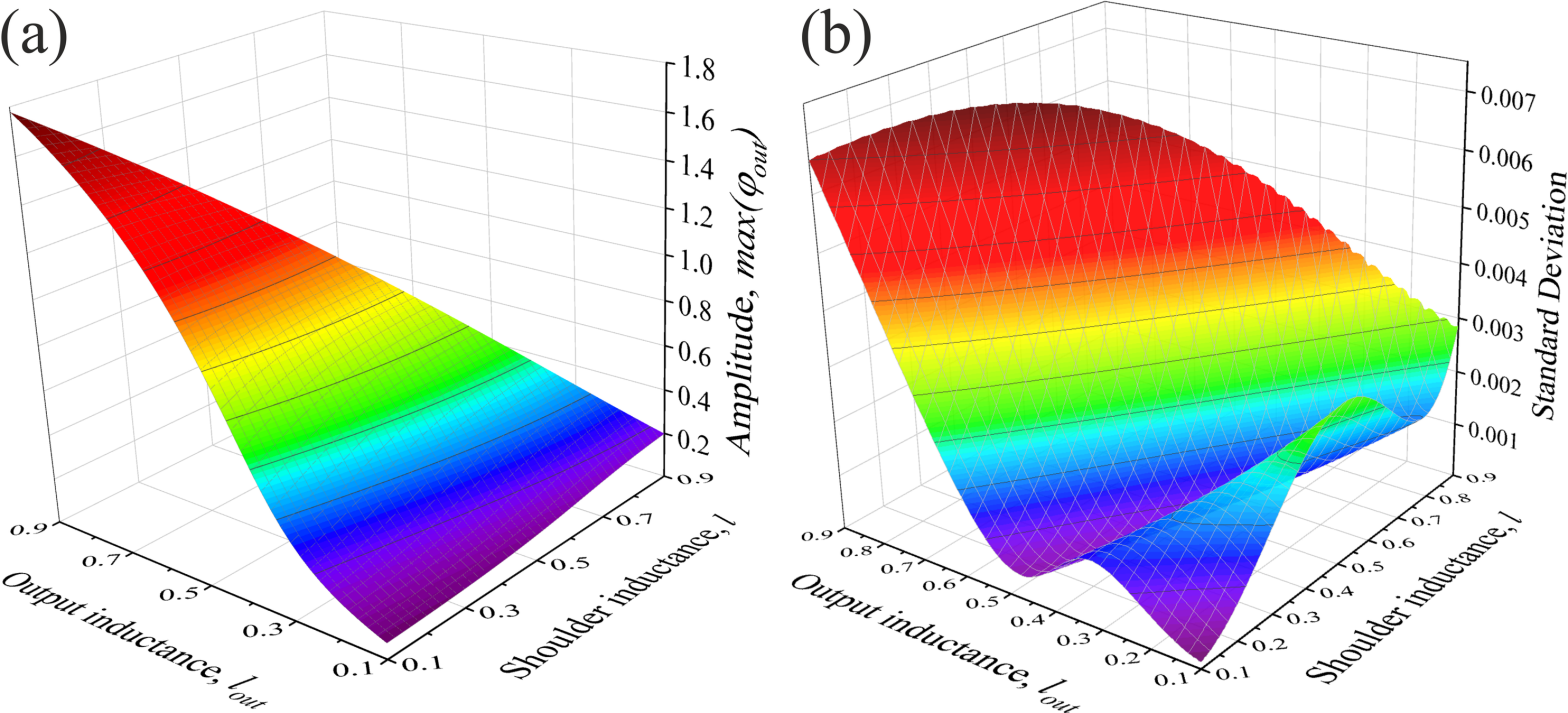
(a) Amplitude of the transfer function and (b) its standard deviation from the Gaussian-like function depending on the inductances *l* and *l*_out_ of the G-cell. The bias flux is equal to 0.05π.

We calculated the standard deviation (SD) of the transfer function from the Gaussian-like function *g*(*x*) with fixed amplitude. The obtained results are presented in the {*l*, *l*_out_} plane. This visualization allows one to find the most proper operating parameters for the considered element. The magnitude of the amplitude of the transfer function is also presented (Figure 3a,b). The optimal values of inductance corresponding to the minimum of SD lies in the hollow of the surface, see Figure 3b. The minimum SD value is reached at *l* = 0.1, *l*_out_ = 0.1. The position of the hollow in Figure 3b could be expressed as (*l*_out_)_SD_ ≈ 0.8 − 0.55(*l*)_SD_. At the same time, for relatively small φ_b_, the transfer function amplitude increases with increase of the output and shoulder inductances, *l*_out_ and *l*. Thus, the choice between the proximity of the transfer function to a Gaussian-like form and the maximization of the response amplitude is determined by the specifics of the network when solving a specific problem. Once again, we emphasize that variations in the parameters of the circuit within a fairly wide range allow one to change the amplitude and width of the activation function, while maintaining its Gaussian-like shape.

**Figure 4:**
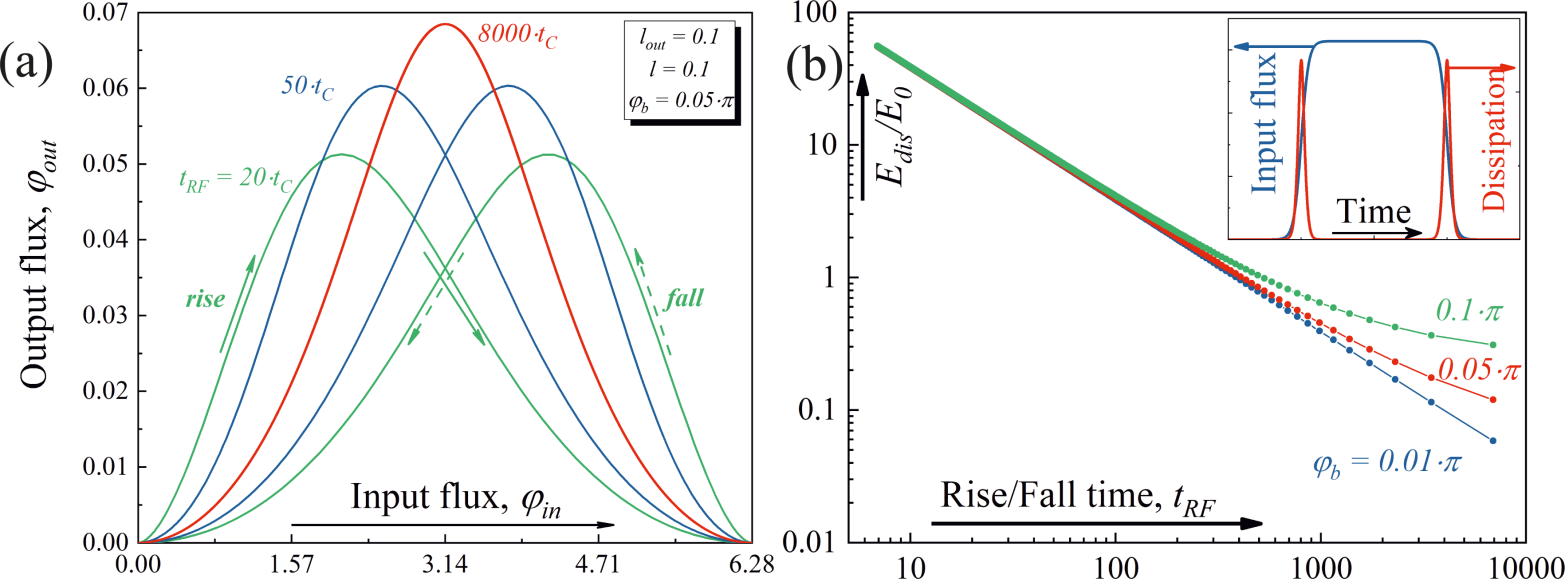
(a) Dynamic transfer function of a Gauss-neuron for a trapezoidal external signal for different values of the rise/fall times of the signal *t*_RF_ and (b) energy dissipation, normalised to the characteristic energy *E*_0_ = Φ_0_*I*_C_/2π, as function of the rise/fall time of the input signal for different bias fluxes: φ_b_ = {0.01, 0.05, 0.1}π. The insert demonstrates the form of temporal dynamic for input flux and dissipation. If the critical current for Josephson junctions *I*_C_ is equal to 100 μA and φ_b_ = 0.05π than *E*_dis_ ≈ 0.01 aJ for *t*_RF_ = 6 ns (corresponds to approx. 1700*t*_C_).

The dynamic transfer functions (i.e., the dependencies of the output current on the time-varying input flux) were also calculated, see Figure 4a. The input magnetic signal is a smoothed trapezoidal function of time with a rise/fall time *t*_RF_, see the inset in Figure 4b. It can be seen that the dynamic activation function of the required type without hysteresis can be obtained with adiabatic operation of the cell (*t*_RF_ up to 8000*t*_C_, where *t*_C_ is the characteristic time for the Josephson junction). The dissipation during the operation of the Gauss-neuron remains small, which justifies classifying the proposed cell as adiabatic (Figure 4b).

### Realization of tunability: adjustable kinetic inductance

For neural networks based on the considered G-neurons, tunable elements with linear current-to-flux transformation (linear inductors) and memory properties are extremely important [50–51]. Tunability of the inductance *l* in Figure 1b allows for an in situ switching between operating modes directly on the chip.

In thin layers of superconductors used to create parts of a neuron, the kinetic inductance is relatively large compared to the geometric one [52]. This is important for us since one can change the kinetic inductance relatively simply by controlling the concentration of superconducting charge carriers (Cooper pairs or superconducting correlations). This approach is the basis of the concept of our tunable in situ Gauss-neuron. A similar idea is used in kinetic inductance devices, which are based on thin superconducting strips [53–54]. They are commonly used for the design of photon detectors and parametric amplifiers. But these devices use nonlinear properties of thin superconducting films at large values of carrying currents comparable to the critical current. However, for our purposes, linear inductors are required. So we consider only the case of a small current in comparison with the depairing current of the superconductor.

In this paper, we propose a tunable kinetic inductance with integrated spin-valve structure [46]. A superconducting spin valve is a device that can control the propagation of the superconducting charge carriers, induced from the superconducting layer via the proximity effect. The typical spin valve [55–57] is a hybrid structure containing at least a pair of ferromagnetic (FM) layers with different coercive forces. Variations in the relative orientation of their magnetizations change the spatial distribution of the superconducting order parameter. In the case of parallel magnetization of the FM layers the Cooper pairs are effectively depairing inside them (closed spin valve). For the antiparallel orientation, the effective exchange energy of the magnetic layers is averaged and suppression of the superconducting order parameter is weaker (open spin valve), providing a propagation of Cooper pairs to the outlying layers of the hybrid structure. The switching between the open and closed states of the valve leads to a noticeable change in the spatial distribution of Cooper pairs. The implementation of a thin superconducting spacer (s) between the FM layers supports the superconducting order parameter and increases the efficiency of the spin valve effect [58]. Here, we propose a development of this approach, allowing one to significantly increase the effective variations in the kinetic inductance.

We study proximity effect and electronic transport in the multilayer hybrid structures in the frame of Usadel equations [59]:


[5]

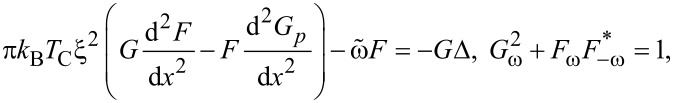




[6] $$\Delta \ln\frac{T}{T_{\text{C}}}+\pi k_{\text{B}}T\sum_{\omega =-\infty }^{\infty }\left(\frac{\Delta }{\left|\omega \right|}-F\right)=0,$$

with Kupriyanov–Lukichev boundary conditions [60],


[7] $$\gamma_{\text{B}}\xi_{\text{l}}\left(\frac{\text{d}F_{\text{l}}}{\text{d}x}-\frac{F_{\text{l}}}{G_{\text{l}}}\frac{\text{d}G_{\text{l}}}{\text{d}x}\right)=F_{\text{r}}-F_{\text{l}}\frac{G_{\text{r}}}{G_{\text{l}}},$$

at the S/FM interfaces. Here *G* and *F* are normal and anomalous Green's functions, Δ is a pair potential (superconducting order parameter), ω = π*k*_B_*T* (2*n* + 1), where *n* is a natural number, *T* is the temperature, *k*_B_ is Boltzmann’s constant, 
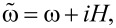
 where *H* is the exchange energy (*H* = 0 in *S* and *N* layers). The indexes “l” and “r” denote the materials at, respectively, the left and right side of an interface, ξ is the coherence length, ρ is the resistivity of the material (in the following, ξ and ρ will also will be mentioned with indexes that denote the layer of these parameters), *T*_C_ is the critical temperature of the superconductor, and γ_B_ = (*R*_B_*A*)/(ρ_l_ξ_l_) is the interface parameter, where *R*_B_*A* is the resistance per square of the interface

The calculated distribution of the anomalous Green function, *F*, permits one to estimate the ability to influence the propagation of the superconducting correlations (screening properties) for the hybrid structure. The spatial distribution of the screening length λ(*x*) directly depends on the proximization of the superconducting order parameter in the system [61–62]:


[8]

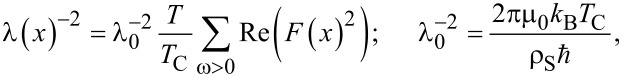



where ρ_S_ is the resistivity of the superconducting material, μ_0_ is the vacuum permeability and ℏ is Planck’s constant. For instance, for a homogeneous niobium film, the estimate for the constant λ_0_ is around 100 nm, while experimentally measured values of the screening length λ at *T* = 4.2 K are around 150 nm. The expression for the kinetic inductance of the structure is directly correlated with screening length [52,63],


[9] $$L_{\text{K}}=\frac{\mu_{0}X}{W}{\left[\int_{0}^{d}{\left[\lambda \left(x\right)\right]}^{-2}\text{d}x\right]}^{-1},$$

where *X* is the length of the strip, *W* is the width, and *d* is the thickness of the multilayer. In our calculations, we assume that the currents in the system are weak, and the structure thickness is much smaller than the screening length.

**Figure 5:**
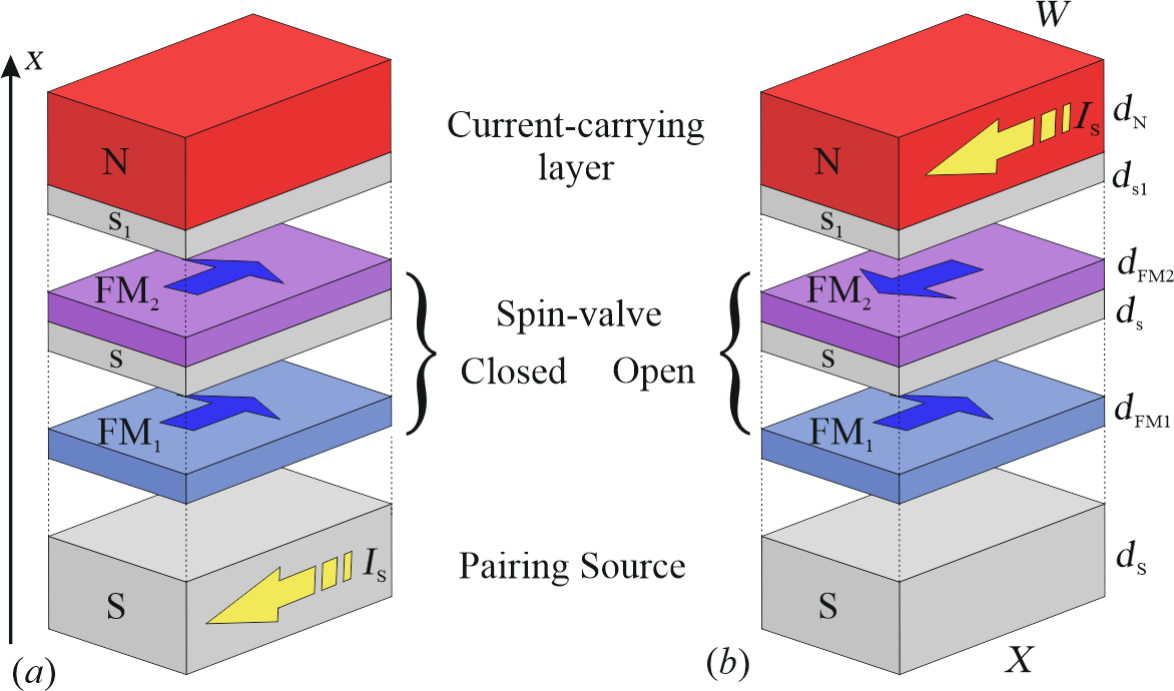
Sketch of the tunable kinetic inductance based on multilayer structure in the (a) closed and (b) open states. Blue solid arrows reveal magnetization orientation of FM_1_ and FM_2_ layers, and dashed yellow arrows demonstrate direction and localization of the supercurrent *I*_S_.

We propose a hybrid structure (see Figure 5) consisting of three parts, namely a pairing source, a spin valve, and a current-carrying layer of normal metal with low-resistivity. The general principle of operation is the following: The pairing source generates Cooper pairs and the spin valve controls their propagation to the layer von low inductance. If the valve is open (Figure 5b), the normal metal is repleted with Cooper pairs, and the biggest part of the supercurrent *I*_S_ is flowing along the structure through the metallic layer (N) with relatively low inductance. In the case of the closed valve (Figure 5a), pairs are locked up in the source layer, and the supercurrent *I*_S_ is limited to this highly inductive part of the structure. The redistribution of the current flowing along the multilayer is associated with a change of the total kinetic inductance.

For a quantitative model, we choose the following components of the structure: The pairing source is a superconductor layer slightly thicker than the critical value at which the pair potential appears. During calculations we suppose its thickness *d*_S_ = 3ξ_S_.

The spin valve can be implemented as a multilayer structure FM_1_–s–FM_2_–s–FM_1_–s–FM_2_ with several ferromagnetic layers FM_1_ and FM_2_ of different thicknesses *d*_FM1,2_ (*d*_FM1_ = 0.15ξ, *d*_FM2_ = 0.1ξ, exchange energy *H* = 100 *k*_B_*T*_C_ in calculations, separated by thin spacers of a superconductor or normal metal (N) (*d_s_* = 0.5ξ for example).

The control of the spin valve is operated by turning the FM layers into states with parallel (P) and antiparallel (AP) mutual orientations of their magnetizations. This process can be realized by application of the finite external magnetic field or by injection of the spin current due to the spin torque effect [56]. For the proposed design of the Gauss-neuron, it is suitable to change magnetizations in the tunable inductance *l* with the control currents in the input circuits, see Figure 1b. Earlier we experimentally demonstrated [58] that for such a control it is sufficient to create a magnetic field strength of the order of 30 Oe. Note that after the control current is turned off, the valve remains in the open/closed state, since the direction of magnetizations in the FM layers is preserved.

The current-carrying layer is a thin strip of normal metal with thickness *d*_N_ = 2ξ and small resistivity ρ_N_ ≪ ρ_S_, which ensures its lower kinetic inductance relative to the rest of the structure. This leads to a flow of the current mostly through this layer in the case of the open valve.

**Figure 6:**
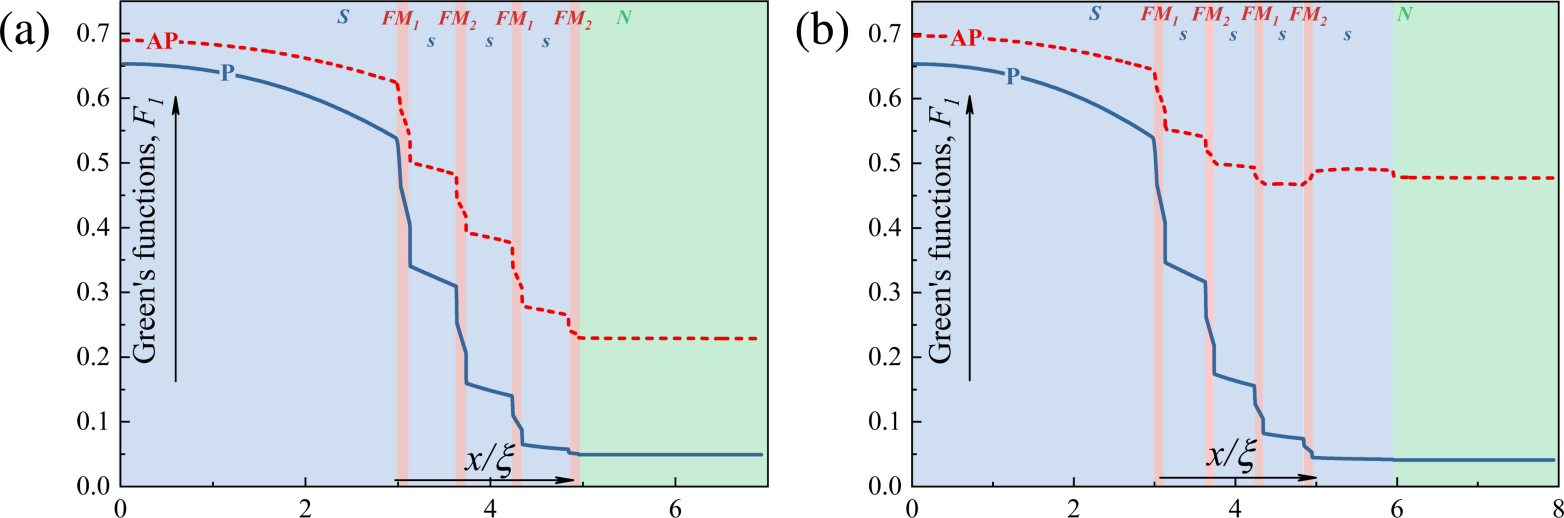
Spatial distribution of the pair amplitude *F* in the hybrid structures (a) S–FM_1_–s–FM_2_–s–FM_1_–s–FM_2_–N without additional s_1_ layer and (b) S–FM_1_–s–FM_2_–s–FM_1_–s–FM_2_–s_1_–N with an additional superconducting layer for parallel (blue solid line) and antiparallel (red dashed line) mutual orientations of magnetization between FM_1_ and FM_2_ layers.

Figure 6a shows the spatial distributions of the pairing amplitude *F*(*x*) in the cross section of this structure for parallel (blue solid line) and antiparallel (red dashed line) orientations of the magnetization of the FM_1_ and FM_2_ layers. The pairing amplitude *F* significantly drops in the spin valve region for both cases. However, the residual level of proximization (value of *F*) in the N layer is five times larger for the AP orientation than for the P orientation.

To enhance the effect, we propose to add an additional superconductor layer s_1_ (Figure 6b). In the case of the closed valve, the s_1_ layer is in the normal state, and the superconducting correlations in the N layer are negligible. If the valve is open, the s_1_ layer goes into a superconducting state with an increase of the pairing amplitude *F* in the N layer up to two times (see Figure 6a).

**Figure 7:**
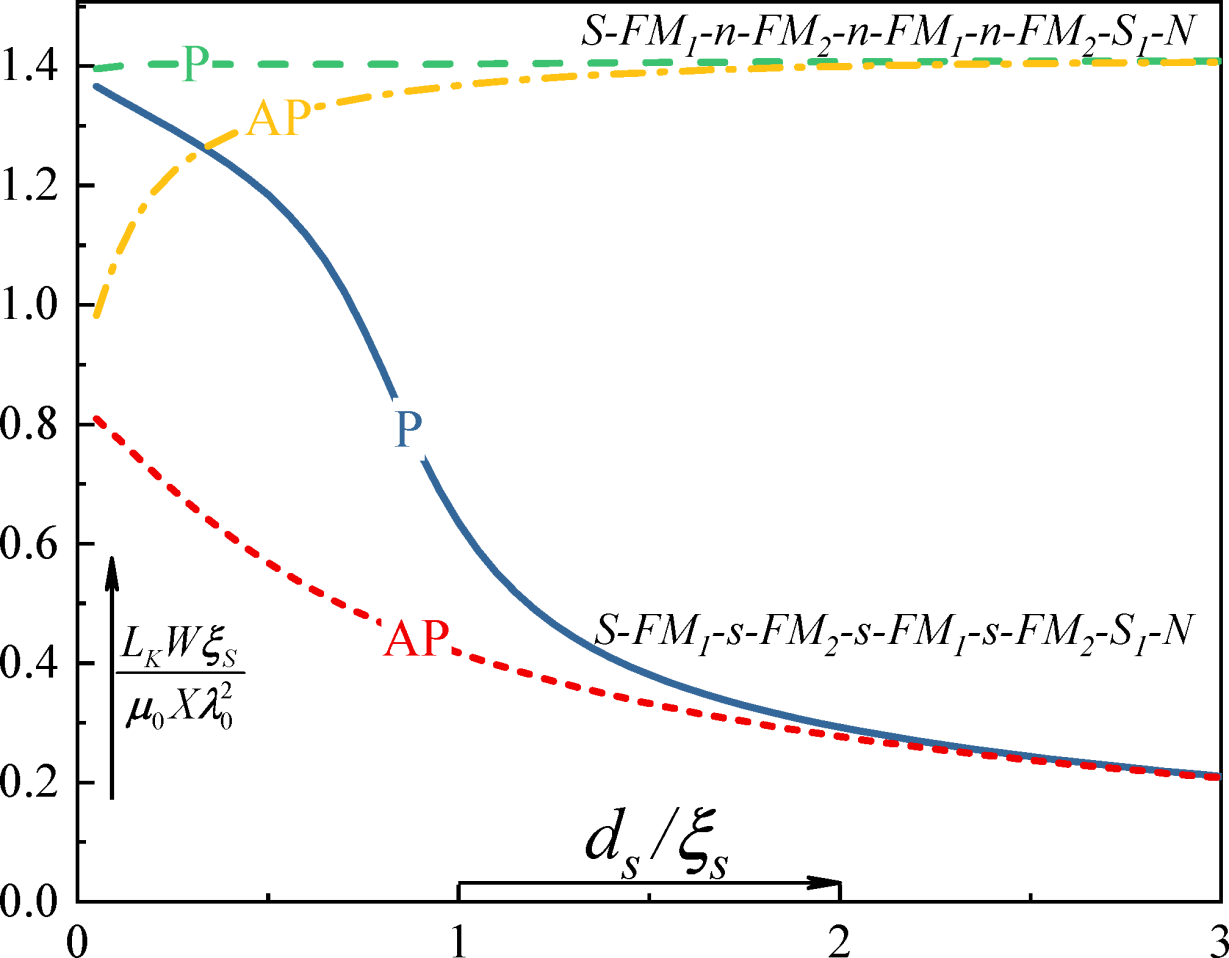
Kinetic inductance of the hybrid structures S–FM_1_–s–FM_2_–s–FM_1_–s–FM_2_–s–N and S–FM_1_–n–FM_2_–n–FM_1_–n–FM_2_–s_1_–N for parallel (dark blue solid line and long dashed green line) and antiparallel (red dashed line and orange dash–dot line) mutual orientations of magnetization between FM_1_ and FM_2_ layers as functions of the spacer thickness.

Figure 7 demonstrates the dependence of the kinetic inductance of the structure shown in Figure 6b versus as a function of the thickness of the intermediate s or n layers. At large thicknesses of the intermediate layers, the valve loses efficiency. In the case of normal spacers, the transition occurs to a completely normal state, where the kinetic inductance of the entire structure coincides with the kinetic inductance of the source layer S. With a large thickness of superconducting spacers s, the valve system also loses efficiency, transferring the entire structure to a completely superconducting state. However, at thicknesses of the order of (0.5…1)ξ, the maximum spin-valve effect appears, and the total kinetic inductance of the structure changes several times during switching between states with parallel and antiparallel magnetization orientations.

We also made some estimates for the quantitative value of the kinetic inductance of the structure shown in Figure 7 based on niobium technology. The inductance of the strip with width *W* = 100 nm, length *X* = 1 μm, and total thickness *d* = 80 nm (this corresponds to the spacer thickness *d*_s_ =5 nm) the estimated kinetic inductance is about 7 pH in the closed state and about 15 pH in the open state. For comparison, the geometric inductance of such a strip is of the order of 1 pH.

## Conclusion

We have considered a basic cell for superconducting signal neurocomputers designed for the fast processing of a group signal with extremely low energy dissipation. It turned out that for this purpose it is possible to modify the previously discussed element of adiabatic superconducting neural networks. The ability to adjust the parameters of the studied Gauss-cell (with Gaussian-like activation function) is very important for in situ switching between operating modes. Using microscopic modeling, we have shown that the desired compact tunable passive element can be implemented in the form of a controllable kinetic inductance. An example is a multilayer structure consisting of a superconducting “source”, a current-carrying layer and a spin valve with at least two magnetic layers with different thicknesses. The proposed tunable inductance does not require suppression of superconductivity in the source layer. In this case, the spin-valve effect determines the efficiency of penetration of superconducting correlations into the current-carrying layer, which is the reason for the change in inductance.

## Appendix

We present the derivation of Equation 2–Equation 4 in the framework of the resistively shunted junction (RSJ) model. A typical approach to obtain the equations of motion for Josephson systems is to write the Kirchhoff and phase constraints. From Figure 1b), it follows:


[10] $$\{\begin{array}{l} i_{1}+i_{2}+i_{\text{out}}=0,\\ \varphi_{1}+i_{1}\cdot l+\varphi_{\text{in}}=i_{\text{out}}\cdot l_{\text{out}}+\varphi_{\text{b}},\\ \varphi_{2}+i_{2}\cdot l-\varphi_{\text{in}}=i_{\text{out}}\cdot l_{\text{out}}+\varphi_{\text{b}}. \end{array}$$

Let us sum up the second and third equations of the system in Equation 10, taking into account the first equation, and dividing the left and right sides by 2:


[11] $$\theta +\frac{i_{1}+i_{2}}{2}\cdot \left(l+2l_{\text{out}}\right)=\varphi_{\text{b}}.$$

As the current through the Josephson junction has a form 
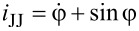
, Equation 11 gives us the first equation of motion (Equation 2) for the Gauss-neuron:


[12]

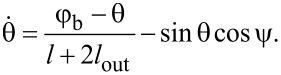



Similar operations should be conducted for the difference between the second and third equations of the system in Equation 10:


[13] $$\psi +\frac{i_{1}-i_{2}}{2}\cdot l=-\varphi_{\text{in}},$$

and the second equation of motion (Equation 3) for the system is obtained:


[14]

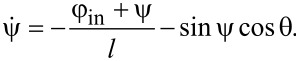



To obtain Equation 4, we have to convert Equation 11 according to the expression *i*_1_ + *i*_2_ = −*i*_out_ = −(φ_out_/*l*_out_):


[15] $$\varphi_{\text{out}}=\frac{2l_{\text{out}}}{l+l_{\text{out}}}\cdot \left(\theta -\varphi_{\text{b}}\right).$$
